# Sequential application of vacuum sealing drainage and antibiotic-loaded bone cement for the successful treatment of a diabetic ischemic foot ulcer: a case report

**DOI:** 10.3389/fendo.2025.1735952

**Published:** 2026-01-16

**Authors:** Xia Feng, Chao Ma, Zhihui Zhang, Lei Xu, Yudong Fang

**Affiliations:** The First Department of Vascular Disease, Shanghai TCM-Integrated Hospital, Shanghai University of Traditional Chinese Medicine, Shanghai, China

**Keywords:** antibiotic delivery, antibiotic-loaded bone cement, case report, diabetic foot ulcer, ischemia, vacuum sealing drainage, wound healing

## Abstract

**Background:**

Diabetic foot ulcers (DFUs), particularly those with ischemic components, present a major therapeutic challenge due to poor perfusion, high infection risk, and delayed wound healing. Conventional treatments often fail to achieve satisfactory outcomes in complex cases. Vacuum sealing drainage (VSD) has shown promise in wound healing by enhancing angiogenesis, stimulating granulation tissue formation, and reducing bacterial colonization while antibiotic-loaded bone cement (ALBC) offers localized, high-concentration antimicrobial delivery. However, the sequential application of these two modalities is rarely reported in ischemic DFUs.

**Case presentation:**

We report the case of a 78-year-old female with type 2 diabetes mellitus who presented with a chronic, infected, ischemic foot ulcer that was unresponsive to standard wound care and systemic antibiotics. Surgical debridement was performed, followed by the application of VSD to enhance granulation tissue formation and maintain negative pressure drainage. Antibiotic-loaded bone cement was subsequently applied to fill the wound cavity and control local infection. Over the subsequent weeks, sequential application of VSD and ALBC resulted in remarkable improvement of the ulcer, ultimately achieving complete wound healing without the need for revascularization or major amputation.

**Conclusion:**

This case demonstrates that the sequential application of VSD and ALBC may offer a synergistic therapeutic strategy for the management of complex diabetic ischemic ulcers. This approach may provide an effective alternative in cases where infection control and wound healing are otherwise difficult to achieve.

## Introduction

Diabetic foot ulcers (DFUs) are a major global health burden, affecting approximately 19%–34% of diabetic patients during their lifetime (1). According to the International Diabetes Federation, the global prevalence of diabetes reached 537 million in 2021, and this number is projected to rise to 783 million by 2045 (2), suggesting that DFUs will continue to be a growing public health issue. DFUs are responsible for over 80% of all non-traumatic lower limb amputations, and once amputation occurs, the 5-year mortality rate can reach up to 70%, which exceeds that of many cancers (1, 3, 4). Among DFUs, ischemic ulcers—caused by peripheral arterial disease (PAD)—are particularly difficult to manage. Ischemia impairs oxygen delivery, delays wound healing, and increases the risk of infection and tissue necrosis (5). Studies have shown that only 24%–40% of DFUs heal within 12 weeks under conventional therapy, cohort and meta-analysis data report significant variation in primary amputation rates—typically ranging from 10% to 30%, with higher rates observed in patients with peripheral arterial disease (4, 6). Current standard treatments include sharp debridement, systemic antibiotics, glycemic control, pressure offloading, and where possible, surgical or endovascular revascularization (7). However, in cases where revascularization is not feasible or infection persists, outcomes are often unsatisfactory.

Vacuum sealing drainage (VSD), or negative pressure wound therapy (NPWT), has demonstrated promising results in managing chronic and infected wounds by promoting angiogenesis, stimulating granulation tissue formation, and reducing bacterial colonization (8). In parallel, antibiotic-loaded bone cement (ALBC) has traditionally been used in orthopedic surgery to treat osteomyelitis or infected prosthetic joints. In the present case, ALBC was used primarily as a local antibiotic delivery system and space-filling material to manage dead space after debridement, while also providing temporary local support to adjacent exposed bone when present, with sustained antibiotic elution over a variable period ranging from days to weeks depending on the cement formulation and antibiotic characteristics (9–11). Currently, ALBC is also widely used in the treatment of diabetic foot ulcers and soft tissue infections (12). Some studies have reported that VSD combined with ALBC can inhibit local inflammation and promote wound healing in diabetic wounds (13). However, the primary purpose of ALBC coverage is to achieve sustained local antibiotic release and enhance antibiotic penetration into surrounding tissues. In contrast, VSD promotes wound healing by continuously draining exudate through negative pressure while maintaining a clean microenvironment and reducing infection risk. When applied simultaneously, VSD may potentially diminish the local concentration of antibiotics released from the bone cement. Therefore, a sequential therapeutic approach—applying VSD and ALBC in succession—demonstrates notable clinical efficacy in promoting wound healing and infection control.

In this case report, we present a patient with a chronic, non-healing ischemic DFU that was successfully managed using a novel combination of VSD and antibiotic-loaded bone cement. This approach may represent a synergistic therapeutic strategy for challenging cases where both ischemia and infection coexist, offering a new perspective on limb salvage techniques in diabetic foot care.

## Case presentation

### Chief complaints

The patient, a 78-year-old female with a 30-year history of type 2 diabetes mellitus presented to our department with a chronic ulcer on the dorsum of her right foot and the hallux, persisting for over 1 month.

### History of present illness

The patient was a 78-year-old woman with a long-standing history of type 2 diabetes mellitus, diagnosed approximately 30 years ago. In the past six months, she gradually developed coldness and numbness in both lower limbs.

About one month before admission, redness, swelling, and pain appeared on the dorsum of her right foot without any identifiable trauma. Despite receiving systemic antibiotics and routine wound care at local hospitals, the lesion continued to worsen. The patient subsequently noted increasing pain, purulent discharge, and areas of black necrotic tissue around the wound.

As the ulcer failed to improve and continued to deteriorate despite prior treatment and the progressive deterioration of the ulcer, she was referred to our hospital for further evaluation and management.

### History of past illness

The patient had a history of poorly controlled blood glucose before ulcer onset, with no significant comorbidities involving the heart, liver, kidneys, or other major organ systems. No significant family history was noted.

### Physical examination

**Figure 1:**
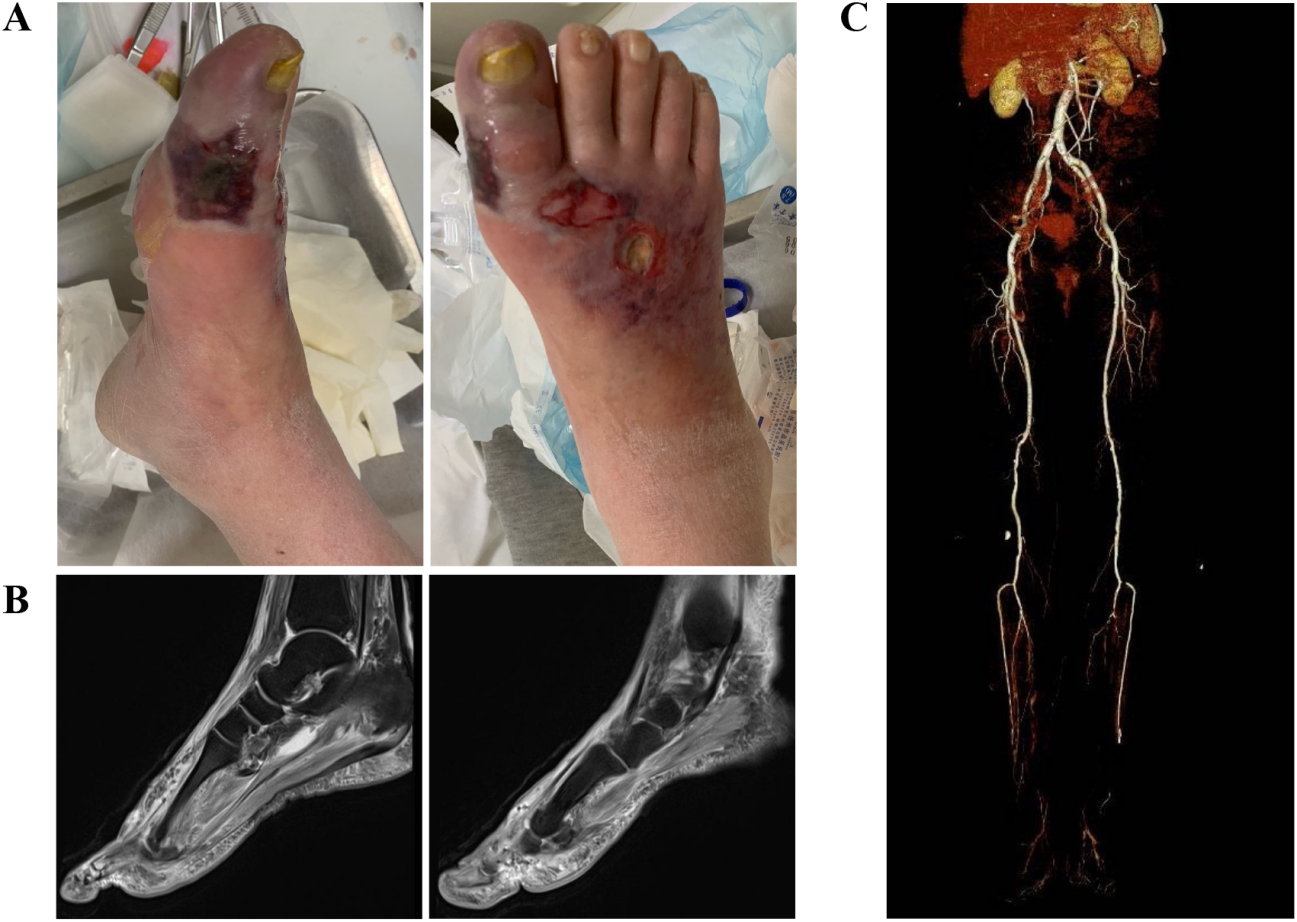
Initial presentation showing a diabetic foot ulcer involving deep tissues and ischemic changes. **(A)** Foot wounds;**(B)** Magnetic resonance imaging (MRI) of the foot; **(C)** Four-dimensional computed tomography angiography (4D-CTA) of the lower extremities;.

Physical examination revealed swelling of the right foot with extensive soft tissue infection extending from the right hallux to the dorsum. The skin was tense with increased turgor and elevated local temperature. Scattered subcutaneous pustules were observed. Following an emergency bedside incision, a large amount of foul-smelling purulent exudate was released. Palpation revealed absent dorsalis pedis and posterior tibial pulses and capillary refill time was delayed, while the popliteal and femoral pulses were present (**Figure 1A**). Ankle-brachial index (ABI) was measured at 0.62, indicating moderate ischemia. This patient presented with sensory neuropathy, which was confirmed by an abnormal response to the 10-g monofilament test.

### Laboratory examinations

Laboratory investigations on admission revealed leukocytosis (white blood cell count, 24.2 × 10^9^/L), elevated C-reactive protein (86 mg/L), an increased erythrocyte sedimentation rate (66 mm/h), elevated interleukin-6 (70.4 pg/mL), and mildly raised procalcitonin (0.258 ng/mL), wound swabs cultured methicillin-sensitive *Staphylococcus aureus* (MSSA). All consistent with active infection.

### Imaging examinations

Magnetic resonance imaging (MRI) of the right foot revealed multifocal bone marrow edema involving the metatarsals, suggestive of osteomyelitis. Associated findings included soft tissue swelling of the forefoot and midfoot, subcutaneous fascial edema, and a dorsal foot ulcer with surrounding tissue breakdown. Joint effusion, consistent with abscess formation, was observed around the first metatarsophalangeal joint (**Figure 1B**).

In addition, four-dimensional computed tomography angiography (4D-CTA) of the lower extremities showed both calcified and non-calcified plaques in the femoral and popliteal arteries bilaterally, accompanied by mild luminal stenosis. The anterior and posterior tibial arteries exhibited both calcified and non-calcified plaques at their origins, with moderate-to-severe stenosis. A segmental occlusion was observed in the distal segment of the right anterior tibial artery (**Figure 1C**). Doppler ultrasonography confirmed reduced arterial flow to the distal extremity (**Table 1**).

**Table 1:** Doppler ultrasound of the lower extremity arteries.

Arterial measurements of the lower extremities		Inner diameter (mm)	Tunica intima	Patterns of blood flow	Rate of blood flow
Right	Common femoral artery	8.8	0.9	Triphasic waveform	104
	Popliteal artery	5.0	0.8	Triphasic waveform	78
	Dorsal artery of foot	1.6	/	Monophasic waveform	17
Left	Common femoral artery	8.6	0.9	Triphasic waveform	115
	Popliteal artery	5.3	0.8	Triphasic waveform	84
	Dorsal artery of foot	1.9	/	Monophasic waveform	31

### Final diagnosis

Based on comprehensive clinical evaluation and diagnostic investigations, the patient was diagnosed with type 2 diabetic foot ulcer (Wagner grade IV), type 2 diabetic peripheral neuropathy, type 2 diabetic peripheral vasculopathy, and peripheral artery disease (PAD).

### Treatment

After completing the necessary examinations, the patient was diagnosed with diabetic foot complicated by lower extremity ischemia.

First, an endocrinologist was consulted to optimize the patient’s glucose-lowering regimen. During hospitalization, blood glucose levels were closely monitored to maintain stable glycemic control.

**Figure 2:**
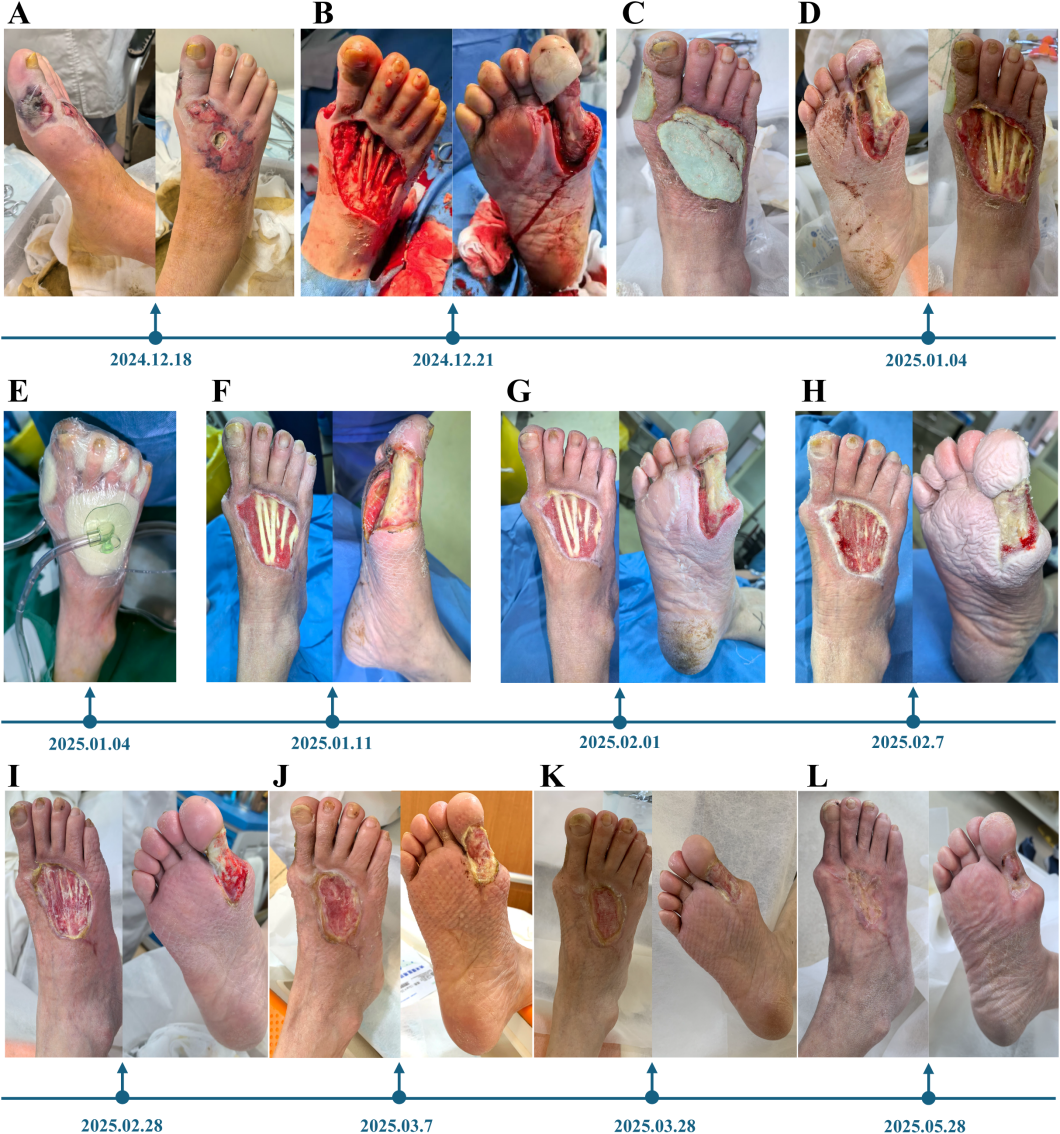
The treatment process of a patient with diabetic ischemic foot ulcer from December 18, 2024 to May 28, 2025. **(A)** Appearance of the affected foot at hospital admission on December 18, 2024; **(B)** Wound condition after the first thorough debridement with complete removal of necrotic and infected tissue on December 21, 2024; **(C)** Appearance of the wound immediately after implantation of antibiotic-loaded bone cement (ALBC) on December 21, 2024; **(D)** Wound status after removal of the ALBC on January 4, 2025; **(E)** Initiation of vacuum sealing drainage (VSD) therapy on January 4, 2025; **(F)** Wound appearance after 1 week of VSD therapy; **(G)** Wound condition 3 weeks after ALBC implantation; **(H)** Wound appearance after 1 week of VSD therapy during the subsequent treatment cycle; **(I)** Wound condition 3 weeks after ALBC implantation during the subsequent treatment cycle; **(J)** Wound appearance after 1 week of VSD therapy during the third treatment cycle; **(K)** Wound condition 3 weeks after ALBC implantation during the third treatment cycle; **(L)** Final wound appearance after completion of the fourth treatment cycle.

Second, under normal circumstances, our standard treatment sequence would involve initial infection control, followed by vascular intervention to restore limb perfusion, and subsequent thorough debridement. However, given the high risk of restenosis following infrapopliteal vascular intervention, the patient declined the procedure after being fully informed of the associated surgical risks. Nevertheless, performing extensive debridement without prior revascularization carries a substantial risk of progressive necrosis. Considering the patient’s preferences and the imaging findings—which revealed no significant stenosis in major arteries but occlusion of small-caliber vessels below the knee with impaired microcirculation—we decided to defer vascular intervention at that time. In light of the patient’s inadequate response to systemic antibiotics and standard dressings, and following comprehensive clinical evaluation, we proceeded with surgical debridement combined with adjuvant therapy. In the perioperative period, systemic infection was managed with intravenous meropenem, and peripheral blood flow was optimized with intravenous argatroban anhydrous and papaverine. Blood glucose levels were concurrently regulated. After thorough removal of all necrotic and infected tissue, extensive exposure of bone and tendon was observed (**Figures 2A, B**). Subsequently, antibiotic-loaded polymethyl methacrylate (PMMA) bone cement was prepared by mixing 3 g of vancomycin powder with 40 g of PMMA cement and was then implanted into the ulcer cavity (**Figure 2C**). In this case, only PMMA bone cement was used. No biodegradable carriers such as calcium phosphate or calcium sulfate cement were applied. Intravenous antibiotics were then discontinued. The bone cement served both as a space-filling material and as a local antibiotic delivery system. Dressing changes were performed every 2–3 days under strict aseptic conditions.

In patients undergoing their initial application of antibiotic-loaded bone cement following debridement, the duration of coverage is tailored based on the wound exudate profile. On day 14, the bone cement was removed, and vacuum sealing drainage (VSD) therapy was initiated with continuous negative pressure at –125 mmHg for 7 days (**Figures 2D, E**). This approach facilitated the removal of exudates, necrotic tissue, and bacteria, reduced edema, increased local blood flow, promoted granulation tissue growth, and minimized external contamination through a closed wound environment. Upon removal of the VSD device, the wound bed demonstrated robust granulation tissue with a healthy red appearance, and partial coverage of the previously exposed tendon and bone was achieved (**Figure 2F**).

Following this, cycles of VSD (1 week) and bone cement implantation (3 weeks) were repeated (**Figures 2G–K**). After four treatment cycles, the exposed tendon and bone were fully covered by healthy granulation tissue (**Figure 2L**). Throughout the treatment period, appropriate offloading was maintained, with initial strict non–weight-bearing during hospitalization followed by the use of a customized postoperative shoe to minimize plantar pressure on the hallux and avoid friction over the dorsal wound.

This treatment course reflects a stepwise and individualized management strategy that integrates repeated debridement, local antibiotic delivery, and intermittent negative pressure wound therapy, offering a structured therapeutic approach for the management of complex diabetic foot ulcers by severe infection, tissue exposure, and even microcirculatory impairment.

### Outcome and follow up

Over the course of treatment, systemic inflammatory markers—including white blood cell count, C-reactive protein, erythrocyte sedimentation rate, and interleukin-6—gradually decreased and returned to normal ranges. The wound showed steady improvement, with healthy granulation tissue progressively covering the previously exposed bone and tendon. Importantly, granulation formation was considered an intermediate milestone rather than the final goal. Continued wound management ultimately resulted in complete epithelialization and full wound closure. The patient regained the ability to ambulate with preserved limb function. At the 4-month follow-up, no recurrence of ulceration or signs of local infection were observed.

## Discussion

Diabetic foot ulcers (DFU), particularly those with ischemic components, represent a severe and complex complication of diabetes mellitus (14). The multifactorial pathogenesis—ranging from peripheral arterial disease and neuropathy to infection—often leads to chronic non-healing wounds and places patients at high risk of lower-extremity amputation (15). In this case, the successful use of combined VSD and ALBC therapy resulted in complete wound closure without the need for major amputation, suggesting a synergistic approach with clinical value.

VSD, also known as negative pressure, wound therapy (NPWT), has been shown to improve local perfusion, reduce bacterial load, and promote granulation tissue formation by generating a controlled sub-atmospheric pressure environment. This technique facilitates drainage of exudates and reduces periwound edema, which are critical for preparing the wound bed for re-epithelialization—especially in ischemic settings (16). Studies have demonstrated that VSD significantly shortens healing time and lowers infection rates in DFUs compared with conventional moist dressings (17). For this patient, continuous negative pressure was preferred over intermittent settings to ensure stable exudate drainage and minimize potential disruption of newly formed granulation tissue (18).

Unlike biodegradable carriers such as calcium sulfate or calcium phosphate, PMMA is non-resorbable and requires planned removal, which allows controlled and sustained local antibiotic delivery, as well as repeated placement during staged wound management (19). The use of antibiotic-loaded polymethyl methacrylate (PMMA) bone cement, widely adopted in orthopedic infections, provides high local concentrations of antibiotics while minimizing systemic toxicity (20). In this patient, vancomycin -impregnated PMMA was placed in the ulcer cavity post-debridement, serving both as an antibacterial depot and a temporary filler to prevent dead space and further contamination. Vancomycin was selected because of its broad activity against Gram-positive organisms commonly implicated in diabetic foot infections and its thermal stability during PMMA polymerization (21). Previous studies have supported the use of vancomycin-impregnated PMMA in the management of infected wounds; however, reports of its application in diabetic foot ulcers remain limited (22).

To our knowledge, the sequential therapeutic approach of VSD with ALBC has not been extensively documented in the management of ischemic DFUs. Given the poor response to prior conventional therapies, neither VSD nor ALBC alone was considered sufficient in this patient. Therefore, a sequential therapeutic strategy combining VSD with antibiotic-loaded polymethyl methacrylate (PMMA) bone cement was adopted. This approach leverages the complementary mechanisms of both modalities: ALBC provides sustained local antimicrobial control and wound stabilization following debridement, while VSD subsequently enhances wound bed preparation by reducing edema and accelerating granulation tissue formation. Such a sequential strategy may offer particular advantages in ischemic diabetic foot ulcers complicated by deep infection, especially in patients for whom revascularization is not feasible or declined. Furthermore, this approach allows for more flexible outpatient management, reducing the risks associated with prolonged hospitalization and bed rest, including pressure injuries, while decreasing economic burden for the patient.

However, this case report has several limitations. First, it represents a single patient experience and lacks comparative or long-term outcome data. Second, although bone cement effectively controlled local infection, it is non-biodegradable and may require removal in other contexts. Further studies with larger cohorts and randomized controlled trials are needed to validate the efficacy and safety of this combined strategy.

In summary, the sequential treatment approach combining VSD and ALBC represents a promising therapeutic option for complex diabetic ischemic ulcers. It highlights the importance of individualized, multidisciplinary treatment strategies in preventing limb loss and improving patient outcomes.

## Conclusion

This case highlights the potential effectiveness of combining VSD with ALBC in the treatment of complex diabetic ischemic foot ulcers. The sequential use of VSD to promote wound healing and ALBC to provide localized infection control may offer a promising therapeutic strategy in patients with poor vascular status and refractory infections. Notably, this method is relatively simple and can be applied as a structured therapeutic approach in various clinical settings, without the need for advanced microsurgical techniques or vascular reconstruction. Early surgical debridement, individualized wound care, and multidisciplinary collaboration remain key to achieving favorable outcomes and avoiding major amputation in this high-risk population. However, further studies with larger sample sizes are warranted to validate the efficacy, safety, and generalizability of this combined approach.

## Data Availability

The original contributions presented in the study are included in the article/supplementary material. Further inquiries can be directed to the corresponding author/s.
